# Blackpatch of Clover, Cause of Slobbers Syndrome: A Review of the Disease and the Pathogen, *Rhizoctonia leguminicola*

**DOI:** 10.3389/fvets.2016.00003

**Published:** 2016-01-27

**Authors:** Isabelle A. Kagan

**Affiliations:** ^1^Forage-Animal Production Research Unit, USDA Agricultural Research Service, Lexington, KY, USA

**Keywords:** *Rhizoctonia leguminicola*, *Trifolium pratense*, slobbers, slaframine, swainsonine

## Abstract

*Rhizoctonia leguminicola* Gough and Elliott is a widely used name for the causal agent of blackpatch disease of red clover (*Trifolium pratense* L.). This fungal pathogen produces alkaloids (slaframine and swainsonine) that affect grazing mammals. Slaframine causes livestock to salivate profusely, and swainsonine causes neurological problems. Although the blackpatch fungus was classified as a *Rhizoctonia* species (phylum Basidiomycota), morphological studies have indicated that it is in the phylum Ascomycota, and sequencing data have indicated that it may be a new genus of ascomycete. The effects of the alkaloids on grazing mammals and their biosynthetic pathways have been extensively studied. In contrast, few studies have been done on management of the disease, which requires a greater understanding of the pathogen. Methods of disease management have included seed treatments and fungicides, but these have not been investigated since the 1950s. Searches for resistant cultivars have been limited. This review summarizes the biological effects and biosynthetic precursors of slaframine and swainsonine. Emphasis is placed on current knowledge about the epidemiology of blackpatch disease and the ecology and taxonomy of the pathogen. Possibilities for future research and disease management efforts are suggested.

## Introduction

*Rhizoctonia leguminicola* Gough and Elliott is a widely used name for the causal agent of blackpatch or black spot disease of red clover (*Trifolium pratense* L.) (1). This fungal pathogen is currently considered most likely to be *Botrytis fabae* Sardiña, according to the database of the U.S. Department of Agriculture (USDA) Systematic Mycology and Microbiology Laboratory (2). Recent work (3) may lead to another reclassification of this pathogen. Blackpatch was first identified on red and white (*Trifolium repens* L.) clover (4–7), but it can also infect other legumes (7–10). On red clover, the disease has caused large losses for producers in the past (5, 6). In addition, alkaloids produced by the pathogen can be harmful to livestock consuming clover hay or pasture, due to excessive salivation (slobbering) and resulting dehydration (11). Few methods exist to manage blackpatch in the field or to mitigate the onset of physiological symptoms of alkaloid consumption. This review briefly discusses the alkaloids of the blackpatch pathogen and the associated problems for livestock. The alkaloids and their effects have been extensively reviewed (11–14), but they provide a context for understanding the characteristics of the pathogen and studies of pathogenicity and disease management. Possible future approaches to disease management are suggested.

## Taxonomy

**Figure 1:**
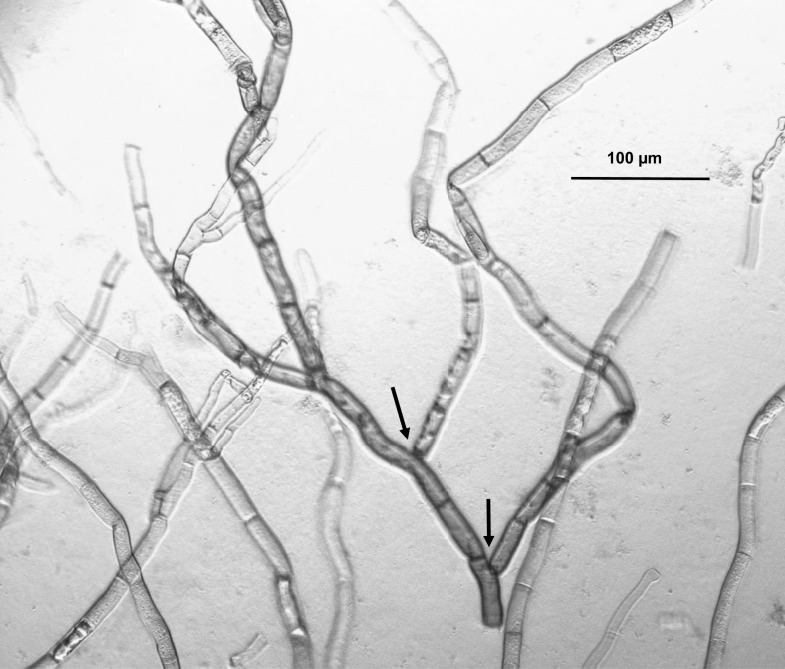
**Light micrograph of *Rhizoctonia leguminicola* mycelium, 100× magnification, grown on red clover agar (120 g/L red clover soaked 4 h in water)**. The dichotomous branching of hyphae and constrictions at the branch points (indicated by arrows) are considered typical of this pathogen.

*R. leguminicola* traits in common with other *Rhizoctonia* species include branched hyphae with septa dividing the hyphae, and an absence of spores (15). Gough and Elliott (1), when naming this fungus, listed the lack of spores, dichotomous branching of hyphae, and constrictions at the branch points (see Figure 1) as reasons for classifying it as a *Rhizoctonia*. Parasitism on roots, detected by Kilpatrick et al. (16) and commonly associated with *Rhizoctonia* species (15), was not detected by Gough and Elliott (1) but not considered critical in the latter’s decision to classify blackpatch as a *Rhizoctonia*. The taxonomy of *R. leguminicola* has been questioned at various times (3, 8, 17). Andersen and Stalpers (17), in a broad study of putative *Rhizoctonia* species in herbaria, examined hyphal morphology and general appearance under a dissecting microscope and sclerotia and infected tissue under a light microscope. The study indicated that the pathogen was *B. fabae* Sardiña, a reclassification making the pathogen an ascomycete instead of a basidiomycete. This is the current name given for the pathogen in the database of the USDA Systematic Mycology and Microbiology Laboratory (SMML) (2). Recent sequencing studies (3) indicate that *R. leguminicola* is indeed an ascomycete, but that it is most closely related to the genera *Pleochaeta* and *Mycocentrospora*. Reclassification as a unique genus, named *Slafractonia*, has been proposed (3). Because neither the genus name nor Mycobank accession number (809882) yield search results on Mycobank at the time of the writing of this review, and the name has not been revised in the SMML database, the pathogen is referred to in this manuscript as “the pathogen,” “blackpatch,” “the blackpatch pathogen,” or “*R. leguminicola*.”

## Alkaloid Production and Physiological Effects

**Figure 2:**
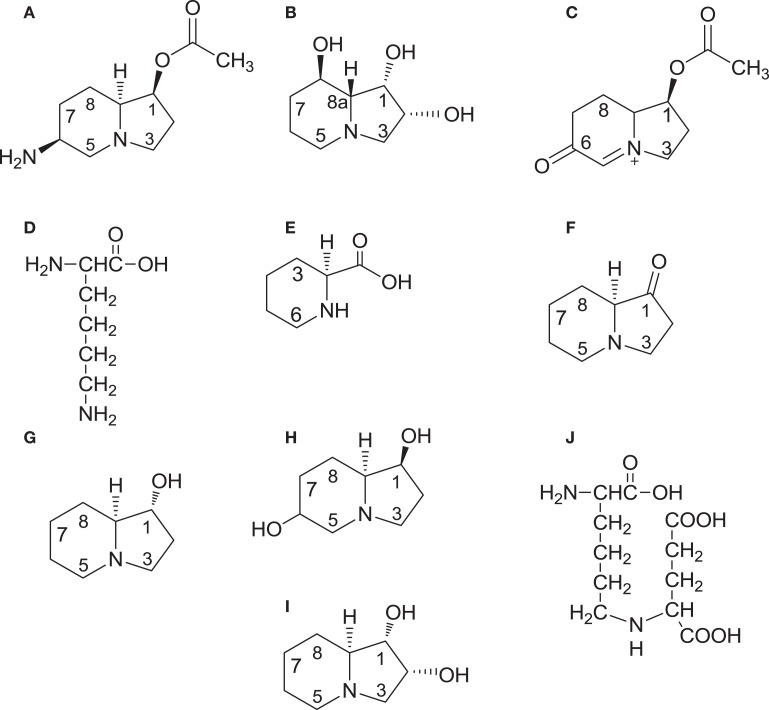
**Structures of slaframine (A), swainsonine (B), the biologically active form of slaframine (C), intermediates [lysine (D); pipecolic acid (E); and 1-oxoindolizidine (F) common to the biosynthetic pathways of both slaframine and swainsonine, swainsonine intermediate *trans*-1-hydroxyindolizidine (G), slaframine intermediate 1,6-dihydroxyindolizidine (H); configuration at C-6 undetermined], swainsonine intermediate *trans*-1,2-dihydroxyindolizidine (I), and common intermediate saccharopine (J)**. On compounds 1G and 1I, *trans* refers to the positions of the hydrogen atoms relative to each other at carbons 1 and 8a.

The presence of the blackpatch pathogen is often signaled by excessive salivation (slobbering) observed in livestock consuming red clover forage (Table 1). The pathogen produces two indolizidine alkaloids: slaframine [(1*S*, 6*S*, 8a*S*)-1-acetoxy-6-aminooctahydroindolizine, Figure 2A] and swainsonine [(1*S*, 2*S*, 8*S*, 8a*R*)-1,2,8-trihydroxyoctahydroindolizine, Figure 2B]. Slaframine, a name derived by Aust et al. (18) from the Old Norse *slafra* (to slaver, a synonym for slobber), is thought to be primarily responsible for the slobbering seen in livestock after consuming infected forages. In forages causing slobbers syndrome, slaframine concentrations varied from 1.5 ppm (9) to 50–100 ppm (19). Broquist (11) reviews the process by which the link was made between slobbering and blackpatch, as well as the work done to purify and characterize the molecule responsible for slobbering. Slaframine has to be converted into an active form in order to have a physiological effect. This requirement was suspected due to the delay between injection of slaframine into the body cavity of rodents and start of salivation (18), suggesting that time was needed to convert slaframine by the liver into an active form. The active form was determined to be a ketoimine (20), with a proposed structure as shown in Figure 2C (the position of the C–N double bond was not confirmed in that study). Slobbering is induced only by the pathogen’s mycelium, and not by the medium in which the pathogen is grown (21). However, legumes in the medium may provide a signal molecule for slaframine production, because slaframine in one study was maximal when cultures were grown on a cold-water extract of red clover (18).

**Table 1:** **Some published reports of outbreaks of “slobbers syndrome**.”

Reference	Location	Animals affected	Associated forage
(19)	North Carolina, USA	Horses	Mixture of second-cutting red clover and orchardgrass hay
(22)	Wisconsin, USA	Dairy cows	Freshly chopped red clover fodder
(23)	Minnesota, USA	Horses	Red clover and alfalfa in pastures (timothy and bromegrass also present)
(24)	Missouri, USA, various counties from 1949 to 1958	Cattle, horses, and sheep	Hay containing red clover (5 of 15 cases involved second-cutting red clover)
(25)	Oklahoma, USA	Horses	Red clover in bermudagrass pasture
(9)	Brazil	Horses	Alfalfa hay
(26)	Brazil	Horse	Red clover in pasture
(27)	Netherlands	Horses	Red clover in pasture

Swainsonine, so called because it was structurally characterized after isolation from the legume *Swainsona canescens* (Darling pea) (28), is the other alkaloid produced by the blackpatch pathogen (29). Swainsonine is also produced by the fungus *Metarhizium anisopliae* (30). It has been identified in other *Swainsona* species (31, 32), as well as from various species of *Ipomoea* (Convolvulaceae) and the legumes *Astragalus* and *Oxytropis*, known as locoweeds (13, 14, 31). Other plant species in which swainsonine has been identified are *Turbina cordata* (Convolvulaceae) and *Sida carpinifolia* (Malvaceae) (31). Swainsonine production in many of these species is strongly correlated with the presence of a fungal endophyte of the genus *Undifilum* in *Astragalus*, *Oxytropis*, and *Swainsona* species (31), and of the order Chaetothyriales in *Ipomoea carnea* (33). The *I. carnea* endophyte (33) and some of the *Undifilum* species (34–36) produce swainsonine in culture, indicating that the presence of swainsonine in plants may be entirely due to production by the plant endophytes.

Biosynthetic studies, conducted by feeding ^14^C-lysine to *R. leguminicola* cultures and recovering ^14^C-slaframine, have demonstrated that slaframine is derived from lysine (Figure 2D) (18, 37), which is converted into pipecolic acid (Figure 2E) (37). The incorporation of ^14^C into slaframine decreased if non-radioactive pipecolic acid was fed along with ^14^C-lysine, an indication that pipecolic acid was a more immediate precursor of slaframine than lysine (37, 38). Pipecolic acid is a precursor of various microbial secondary metabolites (39). Deuterated and tritiated pipecolic acid were incorporated into swainsonine as well as slaframine (40), and ^14^C-acetate and ^14^C-malonate were incorporated into both alkaloids at positions 2 and 3 on the piperidine ring (41). These results indicate that biosynthesis of slaframine and swainsonine proceeds along the same pathway initially, and that the piperidine ring of slaframine and swainsonine is probably formed by nucleophilic attack of malonyl-CoA on the carboxyl carbon of 2E, with subsequent cyclization. The roles of pipecolic acid and malonate in swainsonine biosynthesis are supported by the increased yields of swainsonine obtained in root cultures of *Swainsona galegifolia* fed malonate and pipecolic acid (42).

Feeding tritiated 1-oxoindolizidine (Figure 2F) to *R. leguminicola* cultures yielded tritiated slaframine (37), suggesting that compound 2F is an intermediate. Compound 2F is thought to be the point at which the slaframine and swainsonine biosynthetic pathways diverge because of the opposite configurations of slaframine and swainsonine about carbon 8a (*S* and *R*, respectively) (40). Ketone 2F could conceivably be reduced to *trans*-1-hydroxyindolizidine (Figure 2G), or to *cis*-1-hydroxyindolizidine, with *cis* and *trans* referring to the positions of the hydrogens at carbons 1 and 8a (43). Incubating 1-oxoindolizidine with a crude enzyme extract from *R. leguminicola* resulted in recovery of *cis* and *trans*-1-hydroxyindolizidine, although less of the *trans* form (the likely swainsonine precursor) than of the *cis* form was produced in the incubation (37). When deuterated forms of those hydroxyindolizidines were fed to *R. leguminicola* cultures, the *cis* form was incorporated most efficiently into slaframine, and the *trans* form (Figure 2G) was incorporated most efficiently into swainsonine (43).

In addition to the abovementioned hydroxyindolizidines, some intermediates specific to slaframine or swainsonine biosynthesis have been identified. A 1,6-dihydroxyindolizidine (Figure 2H) was identified in *R. leguminicola* cultures fed deuterated *cis*-1-hydroxyindolizidine (43). The configuration about carbon 6 was not determined (43). Compound 2H was proposed to be a slaframine precursor whose 6-hydroxy group could be oxidized to a carbonyl group and converted into an amino group by transamination (43). Subsequent acetylation at carbon 1 would result in slaframine (43). A tritiated diol (Figure 2I), in addition to tritiated swainsonine, was isolated from separate feeding studies with tritiated *cis* and *trans*-1-hydroxyindolizidine, and it seemed a likely intermediate between compound 2G and swainsonine, given the structure and efficient incorporation into swainsonine when fed (44). Feeding tritiated pipecolic acid to *Astragalus oxyphysus* shoots revealed the presence of tritiated swainsonine and tritiated compounds 2G and 2I, indicating that swainsonine biosynthesis in *A. oxyphysus* shares some of the intermediates of swainsonine biosynthesis in *R. leguminicola* (45).

Subsequent biosynthetic studies with *R. leguminicola* have explored the steps of slaframine and swainsonine biosynthesis between lysine and pipecolic acid. Wickwire et al. (46) demonstrated that the nitrogen in pipecolic acid is derived from the alpha-nitrogen of l-lysine in *R. leguminicola*, and that saccharopine (Figure 2J) is cleaved by a saccharopine oxidase to form delta-1,6-piperideine carboxylic acid (compound 2E with a double bond between N and C-6), a precursor of pipecolic acid. The saccharopine oxidase was purified (47). In *Undifilum oxytropis*, an endophyte of *Oxytropis sericea*, a saccharopine reductase gene was identified (48). Saccharopine reductase catalyzes conversion of alpha-aminoadipic semialdehyde, a lysine precursor, into saccharopine (39). *O. sericea* mutants lacking the saccharopine reductase gene produced more swainsonine and pipecolic acid, and less saccharopine, than wild-type cultures (48). These studies on saccharopine reductase may provide insight on the conversion of saccharopine into slaframine and swainsonine in *R. leguminicola*. The development of a method for proteomics analysis of *R. leguminicola* (49) may facilitate identifying enzymes of slaframine and swainsonine biosynthesis in the blackpatch pathogen.

Because swainsonine causes neurological problems (e.g., staggering, nervousness, and lack of coordination) in livestock that are referred to as locoism (12–14, 50), the first report of swainsonine detection in blackpatch-infected red clover hay suggested that it might contribute to the “slobber syndrome,” a combination of slobbering, feed refusal, bloating, stiffness, diarrhea, weight loss, decreased milk production in dairy cattle, and abortion (51). Violent behavior and lacrimation are sometimes included as symptoms of the slobbers syndrome (12). The role of swainsonine in this array of symptoms is uncertain (12, 52). Croom et al. (12) compared clinical signs in ruminants and horses that were diagnosed with locoism or slobber syndrome, or that were fed purified slaframine and swainsonine. Stiffness, weight loss, and violent behavior were common to locoism, slobber syndrome, and swainsonine ingestion, suggesting that if those clinical signs are observed in slobbering animals, swainsonine may be responsible (12). The presence of unidentified active metabolites may contribute to the slobber syndrome as well, and synergistic effects of swainsonine and slaframine may be involved (12).

The effects of slaframine and swainsonine on livestock depend partly on their stability in blackpatch-infected clover hay and fresh clover. Stability in fresh clover over time does not appear to have been studied. In infected red clover hay stored for 10 months at room temperature, the slaframine content decreased from an initial 50–100 to 7 ppm, a 7- to 14-fold change in concentration (19). Initial swainsonine concentrations were not determined, but the fact that swainsonine (2.5 ppm) was recovered 3 years later from the same hay suggests that it is quite stable, although storage at −50°C may have improved the stability (51). Additional evidence for stability of swainsonine in plants lies in the reportedly stable swainsonine content found over 8 months in root cultures of *S. galegifolia* (42). Also, swainsonine was present in *O. sericea* plants sampled from four geographic locations over a 5-month period, spanning the vegetative to the senescent stages of growth (53). In rangelands, dead locoweed stalks are toxic, demonstrating that swainsonine can persist in dead tissue and continue to be a hazard to grazing livestock (54). In *M. anisopliae* cultures, swainsonine was stable up to 100°C over a pH range of 2–10 (30). Swainsonine reportedly resists autoclaving (55).

Treatment of affected livestock may include removing the infected feed (9, 22, 51) or access to the infected pasture (23, 26). Subsequently, slobbering may cease in 24 h (9, 22, 26, 52) to 3–4 days (23).

## Geographic Distribution and Plant Hosts of Blackpatch

**Figure 3:**
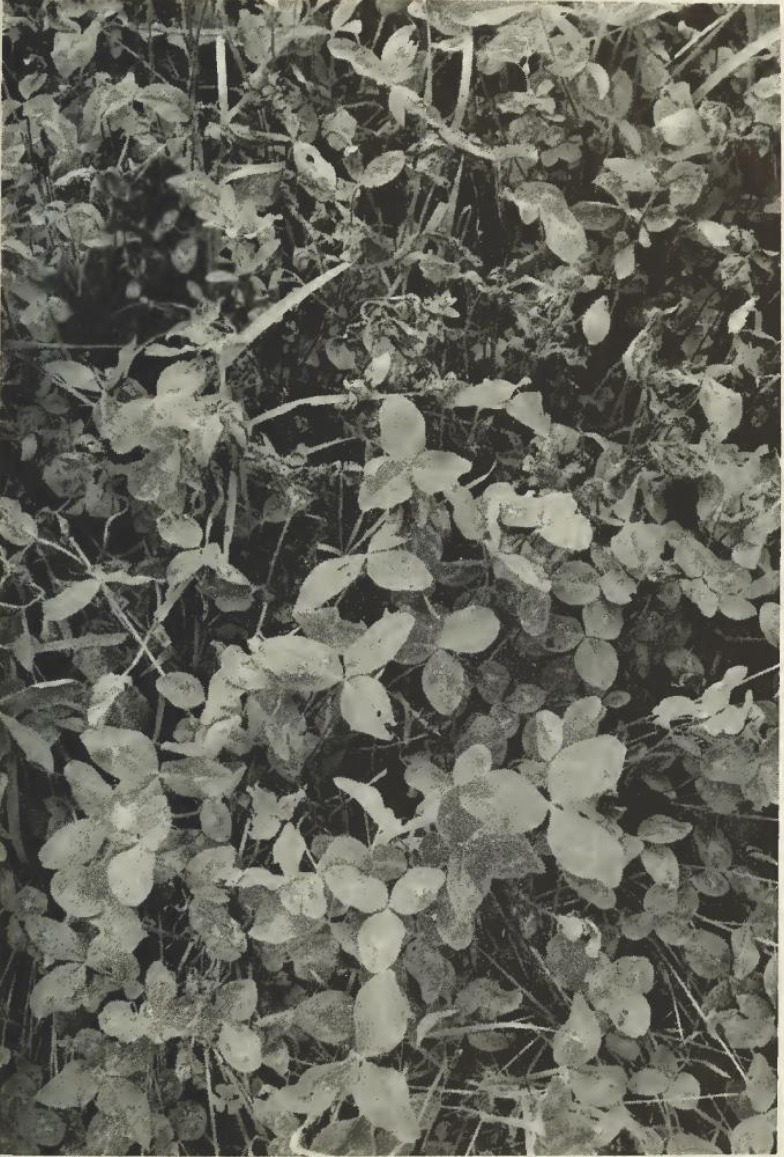
**Blackpatch infection (wilted plants) in a red clover field**. Photo from reference (56), courtesy of West Virginia University Agricultural Extension.

The first report of blackpatch is in a 1933 report of the Kentucky Agricultural Experiment Station. The disease was named for its similarity to brown patch of grasses (4). Leach and Elliott (6) specify that because the mycelium spreads among plants, a “patch of black diseased plants” can develop, as documented by Elliott (56) (Figure 3).

Blackpatch identification methods include isolation of the pathogen from infected plant tissue, with histological analysis (8, 19, 22, 23), administration of infected plant tissue or its extracts to animals to look for slobbering (8, 9, 19), or chromatography to quantify slaframine in extracts (9, 19). Blackpatch has been identified in Canada (8), the midwestern United States (7, 22–24), and the southeastern United States (4–6, 10, 19). It has also been identified in Brazil (9, 26), Japan (12), and the Netherlands (27).

Early reports of blackpatch were based on symptoms on red and white clover (7). In the field, blackpatch also occurs on sainfoin (*Onobrychis viciifolia* Scop.), cicer milkvetch (*Astragalus cicer* Scop.) (8), soybean (*Glycine max* L.) (10), and alfalfa (*Medicago sativa* L.) (9, 57). Host range tests have demonstrated that the pathogen can infect several species of sweet clover (*Melilotus*), and other *Trifolium* species (7, 8). Birdsfoot trefoil (*Lotus corniculatus* L.) has been infected experimentally (8), as have kudzu (*Pueraria thunbergiana* Benth.) and blue lupine (*Lupinus angustifolius* L.) (10).

Because slobbering sometimes occurs in mixed pastures of legumes and grasses (23, 25), and one outbreak was linked to a mixture of red clover and orchardgrass hay (19), it seems possible that the blackpatch pathogen may also infect grasses. Sanderson (22) tested the susceptibility of 11 grass species to four blackpatch isolates. In a humid greenhouse, lesions formed on inoculated *Dactylis glomerata*, *Bromus inermis*, *Panicum virgatum*, *Festuca arundinacea, Phalaris arundinacea*, *Phleum pratense*, *Lolium perenne*, and *Agrostis alba*, and the pathogen was reisolated from each of these grasses. However, lesions were small, unlike the large, coalescing lesions observed on legumes (22). Therefore, it is possible that the pathogen can infect grasses, but that growth is so restricted that no significant amount of inoculum builds up.

## Disease Symptoms and Means of Pathogen Spread

Symptoms associated with blackpatch on red clover include dark brown, often concentric lesions on leaves (7, 8). The size and color of the lesions vary with the host plant (8). On red clover, lesions may also be gray or tan (8). Other symptoms are stem lesions and growth of aerial mycelium over the plant (1, 4). The aerial mycelium is typical of this pathogen (7, 8). It is sometimes difficult to distinguish from red clover pubescence unless examined under a magnifying glass, with the result that the disease may be difficult to detect in the field before it has spread and killed plants (5). Other reports, however, indicate that the disease may be detected from lesions on leaves (7, 23, 26, 27). Blackpatch may be difficult to detect on legume hay, because the mycelial color and texture are sometimes similar to those of cured hay (58). However, Borges et al. (9) found distinct, bright yellow lesions on alfalfa hay. Possibly, the difficulty in detecting the disease depends on the stage at which disease scouting is done. If red clover fields are watched less closely when leaves are emerging than when plants are flowering, early signs of infection on leaves may be overlooked.

The blackpatch pathogen is seedborne but can infect other parts of the plant, such as emerging hypocotyls, and then grow over stems, leaves, and flowers (5). It has been isolated from red clover roots in at least one study (16) but was unable to colonize roots in another (1). Because it does not produce spores, its spread to other red clover plants occurs by the spread of the aerial mycelium to other leaves and stems, which can be quite effective if the healthy and infected seedlings are in close proximity (5, 6). Elliott (56) found that in humid conditions in West Virginia, blackpatch could spread until the majority of a clover field was infected. Transportation of seed can contribute to long-distance dissemination (1).

The ability of blackpatch to serve as field inoculum depends on its ability to overwinter on seed, in soil, or on plant debris. Blackpatch mycelium was viable on seed kept 2 years under “seed storage conditions” (56), presumably at low temperature and humidity. Mycelium on red clover stems was viable after a year of storage at room temperature (1). It survived desiccation for up to 6 months at 20°C, indicating that it might persist in relatively dry regions with moisture from dew (1). The blackpatch pathogen is somewhat sensitive to low temperatures. Mycelium was viable after 30 but not 60 days at −20°C (1), suggesting that prolonged cold temperatures in winter may be detrimental to the pathogen. Because viable mycelium was difficult to find in the field in winter but easily found in the spring, it was suggested that only small amounts of mycelium survive the winter, possibly by overwintering in the crown tissue of red clover (1). Mycelium may also overwinter on seed in the soil (56).

Weather and agricultural practices both play a role in blackpatch outbreaks. Decreases in red clover seed production, indicative of the presence of blackpatch, have been noticed in wet seasons (1). High humidity, which is favorable for red clover growth (56), is sometimes associated with outbreaks of slobbering in livestock (23, 27). An outbreak of slobbers among horses in Brazil was traced to alfalfa hay, which had been harvested at 78% humidity (9). The second cutting of red clover seems to be the biggest source of infected hay (11, 19, 24). Because the second cutting of red clover typically occurs in midsummer, more extensive fungal colonization seems to be associated with higher temperatures, at least in the humid regions in which blackpatch is historically a problem.

## Comparison of Fungal Isolates

Comparing different isolates of a pathogen can give insight into the diversity of the species and possibly help to determine if isolates should be assigned to different species. Sanderson (22) compared four isolates from Canada, North Carolina, and two different Wisconsin counties, based on dry weight accumulation, optimal growth temperature, alkaloid production, type of septa dividing the hyphae, and anastomosis among isolates. Optimal growth temperature did not differ among isolates. Only the two Wisconsin isolates anastomosed with each other (22). The Canadian isolate differed from the other isolates in that it produced no slaframine, accumulated the least dry matter, and had a different type of septa. It also did not anastomose with the other three isolates. Swainsonine was produced by this isolate, although two out of five laboratory cultures of the isolate produced none. These differences suggested that the Canadian isolate might be a different species (22), although it is also possible that this isolate illustrates considerable diversity within the species. DNA sequence information from these four isolates would facilitate determining if they were indeed all the same species.

## Blackpatch Pathogenicity Studies and Their Potential Role in Disease Management

Controlled pathogenicity studies can give insights into potential strategies for managing the pathogen. A culture of the blackpatch pathogen can be obtained from the American Type Culture Collection (ATCC). The pathogen can grow on various types of media. The “salivation factor” (slaframine) was first isolated from fungal cultures grown on medium containing soybean meal, dextrose, calcium carbonate, and corn steep liquor (59). The pathogen has also been grown in stationary liquid culture on red clover infusion media, made by soaking chopped second-cutting red clover hay (200 g/L) in water for 4 h and filtering the infusion through cheesecloth (18). Agar medium made with 100 g/L red clover hay has been used as well (43, 44). Other media used to grow the pathogen include potato-dextrose agar (PDA) (7, 22, 23), malt-yeast broth (8), water agar (5); and soil-extract, oatmeal, and cornmeal agar (7). Cultures on PDA can be stored at −80°C as small squares of mycelium in a 40% glycerol solution of commercial potato dextrose broth. Because mycelium grown on PDA is quite brittle, it can easily be sectioned and deposited into tubes of glycerol media. Such cryostored cultures begin to grow a few days after being briefly thawed in a 25–30°C water bath and transferred to PDA.

In culture, mycelium is hyaline when young and darkens as it ages, becoming green to brown or black (7). Morphology varies on different media. More aerial mycelium was formed on soil extract agar and PDA than on oatmeal agar, and no aerial mycelium was formed on cornmeal agar (7).

**Figure 4:**
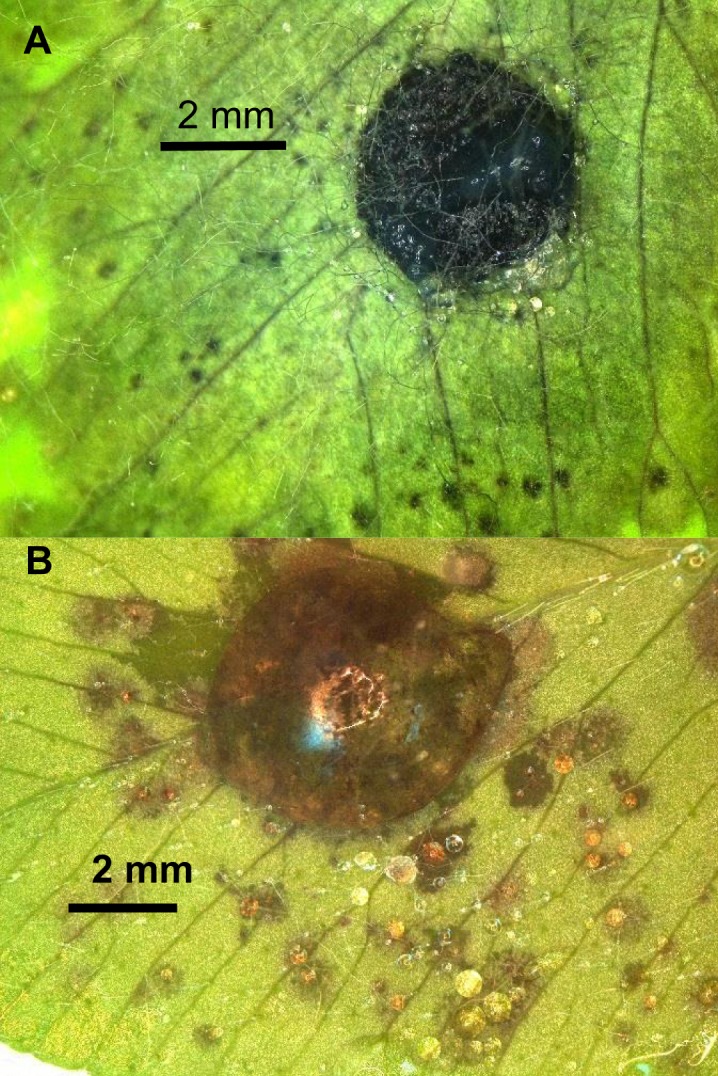
**Symptoms after inoculating a leaflet of the “Kenland” cultivar of red clover with (A) a plug of *R. leguminicola* mycelium from a 13-day-old culture on potato-dextrose agar (PDA), or (B) a 50-μL droplet of homogenized mycelium from a 12-day-old culture on PDA**. Leaflet **(A)** was photographed 66 h after inoculation, and leaflet **(B)** was photographed 49 h after inoculation.

Because the blackpatch pathogen does not produce spores, plants in pathogenicity studies have to be inoculated with the mycelium. Infection can be caused by mycelial plugs set on detached leaves (7, 8), as shown in Figure 4A. Suspensions of mycelial homogenates in water can be sprayed onto whole plants (8) or pipetted onto detached leaves (Figure 4B). Mycelium can also be grown on grain and then dried, crumbled, and sprinkled over plants (22). Measurement of inoculum density is not mentioned in the published descriptions of inoculation methods. Such a measurement could be valuable in pathogenicity studies because the observed symptoms on inoculated plants may depend partly on the inoculum density (60). Concentration of a dried, crumbled grain inoculum could be determined by plating and counting the number of colonies produced from a known mass of inoculum, and a mycelial suspension could be quantified by optical density.

With a reproducible method of inoculating plants and eliciting symptoms, it becomes possible to evaluate cultivars for differences in susceptibility to a pathogen, based on symptom severity. If resistant or partially resistant clover cultivars could be identified in pathogenicity studies, those could be targeted for field trials to determine if the severity of blackpatch, and hence of slobbers outbreaks, could be mitigated with more resistant clover cultivars in pastures. Little information exists on differences in cultivar susceptibility to blackpatch. Susceptibility has been compared among red clover plants collected from areas with a high incidence of blackpatch (1). No resistant cultivars were found in that study, but criteria for resistance were not given. Therefore, it is uncertain if any variations existed in symptom severity. A disease rating scale that evaluates degrees of symptom severity, instead of looking at the presence or absence of symptoms, may facilitate identifying more desirable (less susceptible) cultivars. Differences in percentage of infected leaf area may translate into differences in the extent to which blackpatch spreads in a field. Sanderson (22) compared symptoms on seven red clover cultivars (Norlac, Prosper I, Arlington, Chesapeake, Redman, Redland II, and Pennscott) inoculated in a Wisconsin field with a dried grain inoculum. Symptom severity was scored based on the percentage of necrotic leaf area. One season of data indicated that the Norlac cultivar was more susceptible than the others (22). However, results were inconclusive because no symptoms were observed in the field in the following season, when plants were not reinoculated. These variable field results may indicate that in an initial approach to determining cultivar susceptibility, tightly controlled inoculum applications and environmental conditions (possibly in a growth chamber) are needed.

Reproducible methods of inoculation and symptom elicitation may also permit comparison of alkaloid accumulation in different red clover cultivars. The abovementioned study by Sanderson (22) determined that the Norlac cultivar of red clover, besides being the most susceptible to blackpatch during the season in which symptoms were elicited, had the highest slaframine and swainsonine concentrations in infected tissue. These results suggest that alkaloid production by blackpatch may be affected by host genotype, and that susceptibility may be correlated with alkaloid content in infected plants. In such a case, alkaloid content of cultivars infected by the same isolate may help to identify differences in susceptibility to blackpatch.

## Other Approaches to Disease Management

Fungicide treatments and disease scouting are other potential approaches to blackpatch management. Since blackpatch is seedborne, seed treatments have been studied as a means of control. Benomyl was ineffective in the field (1). Thiram prevented or slowed development of disease in greenhouse studies (5), but it was less effective in the field (1). Several other fungicides (quinolin-8-ol, captan, and zineb) appeared to provide some protection in greenhouse studies, but field data were not provided (1). These studies do not seem to have been continued since the 1950s, suggesting that (a) results were too poor to be worth pursuing, or (b) slobbers syndrome is managed adequately by taking livestock off the suspect hay or pasture, without incurring the expense of fungicide treatments. In the former case, it may be worthwhile to study the efficacy of more recent fungicides, possibly employing foliar sprays or soil treatments with sterol biosynthesis inhibitors like prochloraz, which can inhibit growth of some soilborne red clover fungal pathogens without greatly diminishing growth (61).

A greater understanding of the environmental conditions favoring blackpatch outbreaks may be useful in disease management. Blackpatch is generally associated with humidity, but this understanding is sometimes applied in hindsight, to aid in diagnosing an outbreak of slobbering in livestock (9, 19). If enough information were gathered about locations of infected clover and climatic conditions prior to the finding of the infected clover, such data could possibly be used to model and predict the likelihood of blackpatch outbreaks. Such models are sometimes used to determine when to spray fungicides on certain crops (62), but prediction of blackpatch outbreaks could be used to determine when to keep horses off pasture or look more carefully at red clover hay purchased from areas at risk for an outbreak.

In order to gather information about the distribution and proliferation of blackpatch under different environmental conditions, methods are needed for rapid scouting of blackpatch in the field. Given the difficulty sometimes reported for seeing the pathogen on legumes (5), molecular techniques could facilitate the detection process. Information on the taxonomy and key DNA sequences of the blackpatch pathogen (3) may facilitate designing molecular tools to aid in diagnosing the disease from randomly selected field samples. The specificity of DNA amplification by the polymerase chain reaction (PCR), if done with adequately specific primers, could permit detecting the pathogen in infected material and distinguishing it from other legume pathogens (63). Compared to the standard methods of isolating and detecting slaframine from plant tissue, DNA isolation and PCR would possibly require less time and use fewer hazardous reagents. Slaframine is extracted from plant tissue in methanol (57) or 95% ethanol (19, 22). After concentration and resuspension in water, sometimes followed by partitioning with an organic solvent to remove lipids (22, 57), the aqueous solution is basified and partitioned with chloroform (19, 22) or methylene chloride (57). The organic layer, containing slaframine, is dried, and slaframine is analyzed by thin-layer chromatography (57) or derivatized for gas chromatography (9, 19, 22). High-performance liquid chromatography has been used to analyze slaframine as well (64). Fungal DNA in plants can sometimes be extracted simply by incubating infected plant tissue in microliter volumes of the appropriate extraction and neutralization buffers, due to the specificity of PCR primers for fungal DNA in the extract (65).

## Discussion

Although the blackpatch alkaloids have been studied extensively due to their effects on mammals, the pathogen and methods for its management have been less studied, and little has been done on the latter over the past 30 years. Slobbers syndrome can be managed by removing suspect hay or aspect to a suspect pasture, which may make investigation of the blackpatch pathogen seem unnecessary. However, a better understanding of how to manage blackpatch could be cost-effective for livestock producers who have difficulty affording the cost of replacing hay or finding alternate grazing sites. Red clover producers could benefit from raising cultivars more resistant to blackpatch. Searching for cultivars with resistance to blackpatch and developing a means to predict the likelihood of blackpatch outbreaks, could benefit livestock, livestock owners, and forage producers.

## Author Contributions

IK reviewed the literature and wrote the manuscript.

## Conflict of Interest Statement

The author declares that the research was conducted in the absence of any commercial or financial relationships that could be construed as a potential conflict of interest.
